# Cognitive performance at first episode of psychosis and the relationship with future treatment resistance: Evidence from an international prospective cohort study

**DOI:** 10.1016/j.schres.2023.03.020

**Published:** 2023-05

**Authors:** Edward Millgate, Sophie E. Smart, Antonio F. Pardiñas, Eugenia Kravariti, Olesya Ajnakina, Adrianna P. Kępińska, Ole A. Andreassen, Thomas R.E. Barnes, Domenico Berardi, Benedicto Crespo-Facorro, Giuseppe D'Andrea, Arsime Demjaha, Marta Di Forti, Gillian A. Doody, Laura Kassoumeri, Aziz Ferchiou, Lorenzo Guidi, Eileen M. Joyce, Ornella Lastrina, Ingrid Melle, Baptiste Pignon, Jean-Romain Richard, Carmen Simonsen, Andrei Szöke, Ilaria Tarricone, Andrea Tortelli, Javier Vázquez-Bourgon, Robin M. Murray, James T.R. Walters, James H. MacCabe, The STRATA Consortium

**Affiliations:** a Department of Psychosis Studies, Institute of Psychiatry, Psychology & Neuroscience, King's College London, London, UK; b MRC Centre for Neuropsychiatric Genetics and Genomics, Division of Psychological Medicine and Clinical Neurosciences, Cardiff University, Cardiff, UK; c Department of Biostatistics & Health Informatics, Institute of Psychiatry, Psychology & Neuroscience, King's College London, London, UK; d Department of Behavioural Science and Health, Institute of Epidemiology and Health Care, University College London, London, UK; e Norwegian Centre for Mental Disorders Research (NORMENT), Institute of Clinical Medicine, University of Oslo, Oslo, Norway; f Division of Mental Health and Addiction, Oslo University Hospital, Oslo, Norway; g Division of Psychiatry, Imperial College London, UK; h Department of Biomedical and Neuro-motor Sciences, Psychiatry Unit, Alma Mater Studiorum Università di Bologna, Bologna, Italy; i Department of Psychiatry, Hospital Universitario Virgen del Rocio, IBiS, Universidad de Sevilla, Spain; j Centro de Investigacion en Red Salud Mental (CIBERSAM), Sevilla, Spain; k Social Genetics and Developmental Psychiatry, Institute of Psychiatry, Psychology & Neuroscience, King's College London, London, UK; l South London and Maudsley NHS Mental Health Foundation Trust, London, UK; m Department of Medical Education, University of Nottingham Faculty of Medicine and Health Sciences, Nottingham, UK; n Univ Paris Est Creteil, INSERM, IMRB, Translational Neuropsychiatry, Fondation FondaMental, Creteil, France; o AP-HP, Hôpitaux Universitaires H. Mondor, DMU IMPACT, FHU ADAPT, Creteil, France; p Department of Medical and Surgical Sciences, Bologna Transcultural Psychosomatic Team (BoTPT), Alma Mater Studiorum – University of Bologna, Bologna, Italy; q Department of Clinical and Movement Neuroscience, UCL Queen Square Institute of Neurology, University College London, London, UK; r Early Intervention in Psychosis Advisory Unit for South East Norway (TIPS Sør-Øst), Division of Mental Health and Addiction, Oslo University Hospital, Norway; s Groupe Hospitalier Universitaire Psychiatrie Neurosciences Paris, Pôle Psychiatrie Précarité, Paris, France; t Department of Psychiatry, University Hospital Marques de Valdecilla - Instituto de Investigación Marques de Valdecilla (IDIVAL), Santander, Spain; u Department of Medicine and Psychiatry, School of Medicine, University of Cantabria, Santander, Spain

**Keywords:** Cognition, First episode psychosis, Treatment resistance, Prospective cohort, Schizophrenia, Psychosis

## Abstract

**Background:**

Antipsychotic treatment resistance affects up to a third of individuals with schizophrenia, with recent research finding systematic biological differences between antipsychotic resistant and responsive patients. Our aim was to determine whether cognitive impairment at first episode significantly differs between future antipsychotic responders and resistant cases.

**Methods:**

Analysis of data from seven international cohorts of first-episode psychosis (FEP) with cognitive data at baseline (*N* = 683) and follow-up data on antipsychotic treatment response: 605 treatment responsive and 78 treatment resistant cases. Cognitive measures were grouped into seven cognitive domains based on the pre-existing literature. We ran multiple imputation for missing data and used logistic regression to test for associations between cognitive performance at FEP and treatment resistant status at follow-up.

**Results:**

On average patients who were future classified as treatment resistant reported poorer performance across most cognitive domains at baseline. Univariate logistic regressions showed that antipsychotic treatment resistance cases had significantly poorer IQ/general cognitive functioning at FEP (OR = 0.70, *p* = .003). These findings remained significant after adjusting for additional variables in multivariable analyses (OR = 0.76, *p* = .049).

**Conclusions:**

Although replication in larger studies is required, it appears that deficits in IQ/general cognitive functioning at first episode are associated with future treatment resistance. Cognitive variables may be able to provide further insight into neurodevelopmental factors associated with treatment resistance or act as early predictors of treatment resistance, which could allow prompt identification of refractory illness and timely interventions.

## 1 Introduction

Antipsychotic treatment resistance affects up to a third of individuals with a schizophrenia diagnosis (Siskind et al., 2021). Revised guidelines from the Treatment Response and Resistance in Psychosis (TRRIP) working group (Howes et al., 2017) classify treatment resistance as a lack of symptomatic relief, following at least two or more antipsychotic trials of different drug classes, with adequate adherence, each for at least six weeks' duration, with dosages in at least the mid-point of the licensed therapeutic range. The systematic review from the TRIPP working group identified 42 studies using TRS samples; 21 of these (50 %) did not report operationalised criteria, with 95 % (40 studies) using different criteria (Howes et al., 2017). Therefore this consensus aimed at providing a more standard definition of TRS, something which has not been consistent in previous investigations which may result in heterogeneity across, as well as within, TRS studies (Nucifora et al., 2019). A recent meta-analysis found differences in cognitive performance between individuals with treatment resistant and treatment responsive schizophrenia (Millgate et al., 2022). Unlike those who respond to antipsychotic medication, individuals resistant to medication had cognitive impairments across all cognitive domains, with the greatest deficits observed on verbal memory and language function tasks (Millgate et al., 2022). Cognitive differences between groups have also been observed on verbal intelligence and fluency tasks in first-episode samples with follow-up data to determine future treatment response or resistance (Kravariti et al., 2019), though studies comparing cognitive performance at FEP are limited.

Schizophrenia has been argued to comprise different subgroups based on its neurodevelopmental origins. Compared with adult-onset and late-onset (40–60+ years) subgroups (Murray et al., 1992), early-onset schizophrenia has been associated with greater and earlier brain abnormalities, male sex, earlier use of psychiatric services, and greater negative symptoms and cognitive impairment. Earlier illness onset and male sex have been observed to predict poor therapeutic response to antipsychotic therapy (Carbon and Correll, 2014), and have also been reported as likely predictors of treatment resistance (Smart et al., 2021), suggesting an overlap between the features of neurodevelopmental schizophrenia (Murray et al., 1992), poor therapeutic response, and treatment resistance. It then may be plausible that treatment resistant cases are aetiologically continuous with treatment responsive cases but exhibit a more severe phenotype placing them in a more extreme position on the continuum of neurodevelopmental liability, with differences between treatment responsive and treatment resistant groups becoming more pertinent later in the illness trajectory.

If treatment-resistance is more neurodevelopmentally rooted than treatment responsive schizophrenia, then it stands to reason that it should be identifiable prior to clinical onset. Early detection, or prediction, can aid the initiation of more timely, personalised and appropriate treatment. The gold standard treatment for treatment resistant schizophrenia (TRS) is clozapine (Kane et al., 1988), with this being the only licensed pharmacotherapy for TRS. Early pharmacological intervention with clozapine improving functional outcomes (Üçok et al., 2015), and with response rates of up to 80 % in those treated within the first 2.8 years of illness onset (Yoshimura et al., 2017), but only 30 % for those with delayed treatment (Yoshimura et al., 2017). Despite recommendations in treatment guidelines that delay in starting clozapine for TRS should be avoided, antipsychotic polypharmacy and high dosage are commonly used prior to clozapine, which has been shown to be initiated, on average, 4 years after a diagnosis of TRS (Howes et al., 2012). However, response to clozapine is varied, with an estimated 32 to 39 % of those with a TRS diagnosis also showing non-response to clozapine, (Siskind et al., 2017), being termed ultra-treatment resistant (UTR; Howes et al., 2017). Add-on and augmentation strategies, such as combining clozapine treatment with several antipsychotic agents (e.g. aripiprazole, risperidone and haloperidol), as well as using alternative drugs which target specific symptoms of schizophrenia (e.g. mood stabilisers and antidepressants for negative symptoms) have been recommended for treating this subgroup (Englisch and Zink, 2012), however research concerning this has been largely inconsistent in findings (Leung et al., 2019; Nucifora et al., 2019).

Predictors of treatment resistance will need to be established through large-sample, prospective cohort studies, which capture early impairment. Measures of cognitive performance are easy to administer with relatively low costs, which makes them a feasible candidate for predicting antipsychotic response alongside other established predictors. Indeed, the TRRIP guidelines recommend using clinical (e.g. the Positive And Negative Syndrome Scale, PANSS: Kay et al., 1987) and functional outcome (e.g. Social and Occupational Functioning Assessment Scale, SOFAS; Morosini et al., 2000) rating scales to determine symptomatic improvement, but also highlight the need to clarify individual clinical profiles and treatment resistance based on clinical sub-specifiers, e.g. specific subdomains of positive, negative or cognitive symptoms, rather than overall symptoms (Howes et al., 2017).

Therefore, the aim of this investigation was to determine, in a large international cohort of patients with a first-episode psychosis with a minimum of 1 year follow-up to determine future treatment resistance, whether cognitive function at baseline was significantly associated with future antipsychotic response and whether there was greater impairment in those with a treatment resistant illness. Based on the existing literature (Millgate et al., 2022), we hypothesised that patients with a treatment resistant illness would have more impairment across all cognitive domains than those with a responsive illness, with the largest differences between groups on measures of verbal memory and learning.

## 2 Methods

### 2.1 Sample ascertainment

Datasets were collated as part of Schizophrenia: Treatment Resistance and Therapeutic Advances – Genetics workstream (STRATA-G), a consortium investigating treatment resistance using data from international cohorts of first-episode psychosis patients, which provided demographic, clinical, genetic, neuropsychological and follow-up data (Smart, 2020; Smart et al., 2022). For a dataset to be included, the data had to be prospective, contain first-episode psychosis (FEP) patients with baseline assessments, as well as a minimum of one-year follow-up and genetic data (Smart, 2020; Smart et al., 2022). Cognitive data on at least one domain were available for 683 first-episode participants. Data originated from 7 international cohorts of first episode psychosis with follow-up data to determine treatment resistance states (AESOP, UK; EUGEI & BoFEP, Bologna, Italy; GAP, UK; Istanbul, Turkey; TOP, Oslo, Norway; Paris, France; West London, UK).

Ninety-seven percent (*N* = 618) of this sample had a psychiatric diagnosis at baseline. Patients received a diagnosis of schizophrenia (*N* = 415, 67.2 %), delusional disorder (*N* = 11, 1.78 %), psychosis not specified as schizophrenia (*N* = 109, 17.6 %), schizoaffective disorder (*N* = 50, 8.1 %) and schizophreniform disorder (*N* = 33, 5.34 %). Data for age, age of onset, sex, ethnicity, mode of onset, duration of untreated psychosis, family history of schizophrenia, family history of mental health disorder, body mass index, relationship status, living arrangements, accommodation, employment, years of education, cannabis use, tobacco use, alcohol use, negative and positive symptoms were also collected at baseline.

### 2.2 Definition for treatment response status

Antipsychotic treatment resistance was determined post-hoc using the recommendations from the Treatment Response and Resistance in Psychosis Working Group (TRRIP; Howes et al., 2017). One of the three definitions of treatment resistance were applied in each cohort based on which data was available: i) prescription of or treatment with clozapine during the follow-up period, ii) persistent psychotic symptoms despite treatment with at least two different antipsychotic medications, in at least the mid-point of the licensed therapeutic range and a specified duration (i.e. 6 week's duration with a daily dose of at least 400 mg chlorpromazine equivalents; Leucht et al., 2015), or iii) persistent psychotic symptoms as indicated by symptom measures, and moderate functional impairment despite treatment with two different non-clozapine antipsychotic medications at an acceptable therapeutic dose for at least 6 weeks each. Participants not described by these criteria were classified as antipsychotic treatment responsive. Definitions for each cohort are reported in supplementary material (Table S.1). Four out of the 7 cohorts utilised a definition of TRS which was in line with the recommendations provided by the TRIPP working group (see supplementary material; Table S.1). Two cohorts utilised the second definition for treatment response status. One cohort used treatment of clozapine to define treatment resistance.

### 2.3 Neuropsychological assessment

Cognitive data were collected at baseline i.e. at the first episode of psychosis. In total, 85 individual measures were included in analyses from the 7 cohorts included, spanning 11 cognitive tasks and batteries (Table 1). Data from cognitive tasks were first classified into seven domains by E.M. & E.K., based on each task's underpinning theoretical construct and previous cognitive groupings reported in the literature (Millgate et al., 2022; Fioravanti et al., 2005; Fett et al., 2011; Fatouros-Bergman et al., 2014): i. executive function, ii. attention, working memory & visual-motor/processing speed, iii. IQ/general cognition, iv. visual-spatial memory & learning, v. verbal intelligence & processing, vi. verbal memory & learning, and vii. visual-spatial intelligence & processing. As different cognitive tasks report different scales of measurement, scores were first standardised (z scores) based on the means from the whole sample, where $z=\frac{\left(\mathrm{original score}-\mathrm{whole sample mean}\right)}{\mathrm{whole sample standard deviation}\;}$. These z scores were then averaged across cognitive tasks within a given domain producing a domain-specific composite score. All neuropsychological task batteries and test subsets used are presented in Table 1. A meta-analysis was conducted in STATA 15/SE using the *metan* (Harris et al., 2008) and *metaan* (Kontopantelis and Reeves, 2010) commands to generate effect sizes, as well as determine the extent of heterogeneity in cognitive performance between cohorts. This data is presented in Fig. S.1 and Table S.2 (supplementary material). The means and standard deviations for each domain, using observed data only (i.e. prior to multiple imputation methods), are shown in Table S.3.

**Table 1:** Cognitive domain groupings for all neuropsychological tests and subtests across cohorts at baseline.

Cognitive domain	Neuropsychological tests/battery	Subtest/scores used
Executive function	CANTAB	Intra/Extra-Dimensional Set-Shifting Performance EDS errors
		Tower of London average moves (5 moves) Tower of London total subsequent thinking time (5 moves) Trail Making Test trails B number of errors Trail Making Test trails B time taken in seconds Stockings of Cambridge mean initial thinking time (5 moves) Stockings of Cambridge mean subsequent thinking time (5 moves) Stockings of Cambridge mean problems solved in minimum moves
	Trail Making Test	Trails B number of errors Trails B time taken in seconds
	Verbal fluency	Phonological (Letters) total correct Categorical (Animals/Supermarket) total correct Total errors
Attention, working memory & visual-motor/processing speed	WAIS-R	Digit span task raw total
		Arithmetic raw total
		Digit symbol coding task raw total
	WAIS-III	Digit symbol coding task raw total
		Arithmetic raw total
		Digit Span forward total Digit span backward total Digit span total
	Continuous Performance Test	Reaction time in seconds
	Trail Making Test	Trails A number of errors Trails A time taken in seconds Trails A time taken minus Trail B time taken
	Stroop	Word time taken in seconds Colour time taken in seconds
IQ/General cognition	National adult reading test	Estimated Full Scale IQ
	WAIS-R	Estimated Full Scale IQ
	Wechsler test of adult reading	Estimated Full Scale IQ Estimated Basic Full Scale IQ
Visual-spatial memory & learning	CANTAB	Spatial working memory strategy score Spatial working memory total between errors
		Recognition memory pattern total score Spatial span length
	WAIS-III	Spatial span forward total
		Spatial span backward total
	Wechsler memory scale	Visual recall immediate memory Visual recall delayed memory
Verbal intelligence & processing	WAIS-R	Vocabulary raw total
		Similarities raw total Comprehension raw total
		Information raw total
	WAIS-III	Information raw total
Verbal memory & learning	Rey auditory verbal learning test	Trials 1–5 total
		Trial 7 total
		False positives total
		Recognition total
	Wechsler memory scale	Logical memory total Paired associates learning total
Visual-spatial intelligence & processing	WAIS-R	Block design raw total
		Picture arrangement task raw total
		Picture completion task raw total
	WAIS-III	Block design total
	Raven's progressive matrices	A test total B test total Ravens coloured progressive matrices (CPM) total

*Note. CANTAB* = Cambridge neuropsychological testing automated battery; *EDS* = extradimensional shift; *WAIS-R* = Wechsler adult intelligence scale-revised; *WAIS-III* = Wechsler adult intelligence scale-3rd edition.

### 2.4 Data imputation and analysis

Due to missing data across cognitive domains at baseline (see supplementary material; Table S.3), multiple imputation (Rubin, 2004) methods were applied. Multiple imputation uses distributions in observed data to replace missing data with plausible values (White et al., 2011). Multiple imputation by chained equations (MICE; van Buuren and Oudshoorn, 2000) is a multiple imputation method which assumes that data are Missing At Random, with each variable with missing data regressed on all other variables in the specified imputation model; i.e. a sequential regression multivariate imputation model (Raghunathan et al., 2001). MICE models were executed individually for each cognitive domain using the *mi estimate* command (StataCorp, 2021). This method allows for the combination of imputed datasets following Rubin's rules (Rubin, 2004), with Monte-Carlo error estimates generated using the *mcerror* command (White et al., 2011). 100 imputations and 20 iterations in the burn-in period were set as parameters for this analysis.

We generated the imputed dataset using MICE. The dataset contained data from all cognitive domains, treatment resistance/response status, clozapine use and covariates known to be associated with either cognition or antipsychotic response. Older age (Craik and Bialystok, 2006; Harvey, 2014), the male sex in outpatient samples (and female in inpatients) (Perlick et al., 1992; Han et al., 2012), a longer DUP (Amminger et al., 2002; Chang et al., 2013) and higher positive symptoms and negative symptoms (Addington et al., 1991; Berman et al., 1997) have been reported to impact cognition and cognitive performance in schizophrenia populations. Previous analysis using these data has also demonstrated that the length of follow-up is associated with a greater risk for treatment resistance (Smart et al., 2022). Age at illness onset was included as an additional auxiliary variable (Appendix A and supplementary material; Fig. S.2) to improve the quality of imputed values (von Hippel and Lynch, 2013). This auxiliary variable was associated with missingness in one or more cognitive domains and did not have >75 % missing data itself. The imputed datasets were checked for accuracy through convergence and quantile-quantile plots (supplementary material; Fig. S.3.1-S.3.3 and S.4).

Logistic regressions using these MICE datasets (containing both observed and newly imputed values) were performed to predict treatment resistance/response status using baseline cognitive performance, in each cognitive domain as predictors. Unadjusted analyses were conducted, then multivariate models controlling for age, gender, cohort, length of follow-up, duration of untreated psychosis (DUP), positive symptoms (assessed by the SAPS) and negative symptoms (assessed by the SANS).

### 2.5 Sensitivity analyses

In order to determine the effects of our definitions used for treatment resistance as well as our sample inclusion criteria for diagnosis, sensitivity analyses were applied. Logistic regressions following multiple imputation was conducted on a restricted sample which had received a diagnosis of schizophrenia (*N* = 415), i.e. first episode schizophrenia (supplementary material: Table S.4, Table S.5) In addition, logistic regression models were used to predict clozapine use (*N* = 618) during the period of follow-up (supplementary material: Table S.6, Table S.7), a similar criteria used in previous population studies of treatment resistance (Wimberley et al., 2016).

## 3 Results

Table 2 shows the proportion of treatment responsive and treatment resistant cases in the sample, stratified by cohort.

**Table 2:** Proportion of treatment resistant and treatment responsive cases per cohort.

Cohort	Treatment responsive N (%)	Treatment resistant N (%)	Total
AESOP	55 (70 %)	24 (30 %)	79
Bologna	22 (85 %)	4 (15 %)	26
GAP	70 (77 %)	21 (23 %)	91
Istanbul	35 (81 %)	8 (19 %)	43
Oslo	133 (99 %)	2 (1 %)	135
Paris	22 (82 %)	5 (18 %)	27
West London	268 (95 %)	14 (5 %)	282
Total	605 (89 %)	78 (11 %)	683

### 3.1 Demographic and clinical characteristics

Table 3 illustrates the baseline demographic and clinical characteristics of both antipsychotic responder and antipsychotic resistant groups, with these groups determined from follow-up data (see Smart et al., 2022). Treatment resistant participants were younger, with an earlier age of onset, a longer length of follow-up, and had higher clinical ratings of negative symptoms in comparison to treatment responders.

**Table 3:** Demographic and clinical characteristics of patients with antipsychotic responsive and antipsychotic resistant illness.

Demographic/clinical variable	Proportion of missingness (%)	Responder			Treatment resistant		
		N	Mean/%	SD	N	Mean/%	SD
Age	0 %	605	27.25	8.66	78	24.64	7.58
Age of onset	1.02 %	599	25.62	8.47	77	23.63	7.52
Sex	0.73 %	Male = 400 Female = 200	Male = 67 % Female = 33 %	–	Male = 53 Female = 25	Male = 68 % Female = 32 %	–
Duration of untreated psychosis (days)	19.33 %	504	534.11	1038.47	47	543.80	922.67
Length of follow-up (days)	6.15 %	563	1326.20	1038.33	78	2219.18	1330.34
SAPS	52.42 %	303	10.84	4.00	22	9.50	4.35
SANS	54.17 %	292	9.59	6.42	21	12.01	7.05
Ethnicity	45.53 %	European = 206 African = 53 Asian/Mixed = 54	European =65.8 % African = 16.9 % Asian/Mixed = 17.3 %	–	European = 29 African = 21 Asian/Mixed = 9	European = 49.2 % African = 35.6 % Asian/Mixed = 15.3 %	–
Mode of onset	70.86 %	Abrupt = 13 <6 months = 67 Within 6 months = 67 >6 months = 27	Abrupt = 7 % <6 months = 38.5 % Within 6 months = 38.5 % >6 months = 15.5 %	–	Abrupt = 4 <6 months = 6 Within 6 months = 12 >6 months = 3	Abrupt = 16.0 % <6 months = 24.0 % Within 6 months = 48.0 % >6 months = 12.0 %	–

Note. SAPS = Scale for the Assessment of Positive Symptoms; SANS = Scale for the Assessment of Negative Symptoms.

### 3.2 Neuropsychological results

Results from the meta-analysis comparing standardised baseline cognitive performance between treatment responders and treatment resistant samples across cohorts observed small effect sizes across cognitive domains (supplementary material: Fig. S.1, Table S.2). Heterogeneity estimates, as determined by Cochrane Q values, observed no significant presence of heterogeneity in cognitive performance between cohorts (supplementary material: Table S.2).

### 3.3 Association analyses

Following MICE, in unadjusted logistic regressions (Table 4) treatment resistance was significantly associated with poorer performance at first episode on IQ/general cognition, executive function and verbal intelligence and processing domains. After adjusting for age, gender, cohort, DUP, length of follow-up and positive and negative symptoms, only the association between impaired performance in IQ/general cognition and treatment resistance remained significant. In univariable models, one standard deviation (SD) increase in performance on the standardised (z score), relative to the whole sample, IQ/general cognitive functioning was associated with reduced odds for being termed treatment resistant (OR = 0.70, *p* = .003). Small changes to odds ratios were observed in this domain in the adjusted model.

**Table 4:** Logistic regression models for unadjusted and adjusted models comparing the relationship between baseline cognitive performance and treatment resistance.

	Cognitive data sample size prior multiple imputation		Cognitive data sample size following multiple imputation		Unadjusted					Adjusted				
Domain	N responder	N treatment resistant	N responder	N treatment resistant	β	SE	95%CI	OR	*P*-value	β	SE	95%CI	OR	P-value
Executive function	340	40	605	78	−0.12	0.24	−0.59; 0.34	0.89	0.611	−0.03	0.26	−0.54; 0.48	0.97	0.901
Attention, working memory & visual-motor/processing speed	356	45	605	78	−0.22	0.17	−0.55; 0.12	0.81	0.208	−0.18	0.20	−0.57; 0.22	0.84	0.379
IQ/General cognition	553	71	605	78	−0.35	0.12	−0.59; −0.12	0.70	0.003	−0.28	0.14	−0.55; −0.002	0.76	0.049
Visual-spatial memory & learning	312	28	605	78	0.30	0.27	−0.23; 0.83	1.35	0.262	0.10	0.28	−0.46; 0.66	1.10	0.737
Verbal intelligence & processing	270	24	605	78	−0.39	0.16	−0.71; −0.08	0.66	0.014	−0.31	0.19	−0.67; 0.07	0.74	0.107
Verbal memory & learning	297	32	605	78	−0.27	0.22	−0.70; 0.15	0.76	0.207	−0.19	0.23	−0.65; 0.27	0.83	0.416
Visual-spatial intelligence & processing	317	38	605	78	−0.10	0.16	−0.41; 0.20	0.90	0.503	0.02	0.20	−0.37; 0.42	1.02	0.908

Adjusted: adjusted for age, gender, cohort, duration of untreated psychosis, length of follow-up, SAPS & SANS.

Note. OR = Odds Ratio; CIs = confidence intervals.

### 3.4 Sensitivity analyses

Restricting our sample to only those with a schizophrenia diagnosis observed no significant predictive relationships between standardised baseline cognitive performance and future antipsychotic treatment resistance or response status in unadjusted and adjusted logistic regression models following imputations (supplementary material: Table S.5). A similar pattern of results was observed when using clozapine use within the follow-up period to classify response status (supplementary material: Table S.7).

## 4 Discussion

In the present study, using longitudinal data from seven international cohorts of first episode psychosis, our results showed that participants who later proved to be resistant to antipsychotic treatment had worse baseline IQ/general cognitive performance in comparison to those with a responsive illness. These cognitive estimates were based off standardised cognitive scores generated from the whole sample performance (i.e. these results are relative to other individuals with first episode psychosis). Due to missingness in data across cohorts, multiple imputation methods were used. Logistic regression following imputation found a significant relationship in unadjusted models for IQ/general cognition and verbal intelligence and learning, with IQ/general cognition tasks maintaining this relationship in adjusted logistic models.

Against a picture of known heterogeneous deficits in schizophrenia, this investigation sought to identify further nuanced differences within sub-groups of patients who were classified as responding, or not responding, to antipsychotic medication at follow-up. Based on previously reported neuropsychological differences between treatment-responsive and treatment-resistant schizophrenia, largely based on findings from cross-sectional studies (*dl* = −0.53; Millgate et al., 2022), we hypothesised that greater deficits in verbal memory and learning performance at FEP would be observed in the future treatment resistant group in comparison to other cognitive domains. With this meta-analysis including mostly cross-sectional studies, sample chronicity and long-term medication effects could have influenced the findings in comparison to a first episode sample. In our sample the association between verbal memory and learning and treatment resistance was not observed, with verbal intelligence deficits only being associated with response status in unadjusted logistic regression models, and only measures of general intelligence observing a significant relationship between standardised baseline cognitive performance and future follow-up response status.

Despite our imputation models imputing well, as evidence by convergence and quantile-quantile plots (supplementary material: Figs. 3.1–4), the IQ/general cognitive domain was the only composite domain to receive data from all 7 cohorts included in the study (supplementary material: Fig. S.1), had substantially less missing data (8.64 %), and attained the largest sample size (*N* = 624) than other domains. Due to this it is possible that this domain captured a wider range of performance in samples, improving the placeholders used in imputation models. In contrast our imputation model may be limited in their ability to capture the true variance and differences in scores between responder groups in other cognitive domains because they were imputed from a limited number of datapoints.

Similarly, it is possible that impairments in IQ/general cognitive function may provide a more holistic scope of cognitive impairment in the first episode, whereas research has observed deficits in other domains, such as verbal memory and learning, to become more exaggerated later in the course of the illness, particularly for those who experience psychotic relapse or non-remission of symptoms (Barder et al., 2013). In a recent population-based, case-control study, comparing cognitive trajectories in FEP with healthy controls over a 10-year period (Zanelli et al., 2019), deficits in measures of executive function, processing speed and visuospatial ability were apparent at first episode and remained stable over time. In contrast, cognitive decline was observed in measures of verbal knowledge, memory, and full-scale IQ suggesting that this may reflect a distinction in decline between measures of executive function and processing speed versus memory and verbal knowledge. Measures such as verbal memory have routinely reported the largest effect sizes in comparison to controls in first episode (Mesholam-Gately et al., 2009), drug-naïve (Fatouros-Bergman et al., 2014) and chronic (Heinrichs and Zakzanis, 1998) samples of schizophrenia, as well as between treatment resistant and treatment responsive samples (Millgate et al., 2022). If we then understand treatment resistance to be etiologically continuous with treatment responsive schizophrenia but present a more exaggerated neurodevelopmental profile, the larger deficits we have observed in IQ/general cognitive function may support the claim of specific declines in certain cognitive domains over the disease timeline.

However, an alternative explanation may be that there is an underlying cognitive factor (‘g’) which explains the variance between these cognitive domains (Dickinson et al., 2011), suggesting a greater generalised cognitive impairment at first episode in future treatment resistant patients versus treatment responders, particularly when cognitive performance scores are then standardised to the mean of the whole sample. Due to a lack of normative healthy control data in the sample, cognitive scores were standardised to the whole sample performance, meaning that our findings reflect a deficit in performance relative to other individuals with first episode psychosis. This would explain why effect sizes from meta-analyses were generally small (supplementary material: Table S. 2) in comparison to previous investigations comparing performance against healthy controls (e.g. Heinrichs and Zakzanis, 1998; Mesholam-Gately et al., 2009). Unfortunately, it was not possible to generate an overall composite general ability cognitive domain due to missingness of data and a reduced number of variables to specify in multiple imputation models.

With the results of this study finding a significant association between worse performance at first episode in IQ/general cognition and receiving a treatment resistant classification in the future, it is important to consider the clinical relevance of this finding. While current neuropsychological batteries are helpful to practitioners in providing a detailed picture of a patient's neuropsychological profile, these may not always be beneficial to routine practice. The American Psychological Association's Working Group on Screening and Assessment have provided guidelines for determining the appropriateness of a neuropsychological measure for cognitive screening within a clinical setting (American Psychological Association, 2014). The guidelines are as follows: i. provide identification for those at high risk for impairment, ii. sensitive enough to identify those who need further review, iii. Brief and narrow in scope, iv. can be administered at routine visits, v. can be administered by support staff or clinicians electronically and vi. can be used to monitor progress and outcomes (Roebuck-Spencer et al., 2017). Currently, it has been argued that the use of cognitive, in addition to clinical, markers in the prediction of psychosis has been argued to add nonreductive predictive value (Studerus et al., 2016).

Therefore, if differences in cognitive performance between treatment responders and non-responders can detect meaningful specificity and sensitivity in the first-episode, this could allow clinicians to appropriately determine who may benefit from an early course of clozapine, or add-on/augmentation strategies with other mood stabilising or antipsychotic agents (e.g. polypharmacy). Not only could this help improve patient outcomes for those with a treatment resistant illness, with better outcomes observed in patients who initiate clozapine within the first 3 years (Yoshimura et al., 2017), but also can help reduce the economic and medical costs of treatment resistance, estimated as being 3 to 11 times larger than those who respond to first-line antipsychotic medication (Kennedy et al., 2014). This highlights a need to improve the standardization and validation of cognitive tasks in the prediction of schizophrenia early in its clinical course, which could then be extended to further explore prediction in treatment resistant schizophrenia, which could help improve timely and appropriate pharmacological intervention.

### 4.1 Limitations

In our main analyses, this study used samples of participants with a FEP rather than restricting the diagnosis to schizophrenia. The treatment resistant group had a higher proportion of cases with schizophrenia (73 %) than the responder group (59 %). A recent meta-analysis has suggested that at first episode there is a high level of misclassification of schizophrenia with other psychotic disorders, warranting broader diagnostic inclusion criteria (Fusar-Poli et al., 2016), and so we felt justified including a broader diagnosis inclusion criteria for participants in to improve power. This method is also comparable to other investigations predicting treatment resistance (Legge et al., 2020) An arguably underpowered sensitivity analysis restricted to only samples with a schizophrenia diagnosis (treatment responsive schizophrenia *N* = 358; treatment resistant schizophrenia *N* = 57) was unable to replicate the associations reported in main analyses in both unadjusted and adjusted models (supplementary material; Table S.4 & Table S.5).

The rate of treatment resistance in this sample containing cognitive data in at least one cognitive domain was 11.4 %. This proportion of treatment resistance is smaller than the total sample (17 %; as reported in Smart et al., 2022), as well as the current rates of treatment resistance from first-episode cohorts (20–30 %; Stokes et al., 2020; Siskind et al., 2021). Due to the inclusion of samples with pre-collected first episode psychosis data from different cohorts and time points, of which did not have treatment resistance as an original follow-up outcome, it is possible that our sample may be biased toward those with a treatment resistant illness who had more preserved cognition than those who did not provide cognitive assessments.

Likewise, it is also possible that due to the missingness of data across cognitive domains the estimates generated from MICE analyses may lack representativeness of the sample population. As the contribution of cognitive data from each cohort was relatively small across participants, it is possible that patients who were able to provide cognitive data may have been less unwell, more willing to participate, and had less cognitive symptoms permitting them to complete the cognitive tasks. Therefore it is possible that our MICE method, which imputed the data well based on the original cohort data, may be capturing a specific sub-group within the sample which has the affinity to complete and provide data on cognitive performance. This may have resulted in a more homogenous cognitive profile between treatment resistant and treatment responders in this sample. Similarly without a healthy control comparison group, these results could not be compared against a sample of normal cognitive function.

Inherent in all studies of treatment resistance, it is also possible that some cases in the treatment responder group could actually have been treatment resistant but did not accept or tolerate clozapine and were therefore misclassified. Similarly, it is possible that some individuals in the responder group could have developed treatment resistance after the study follow-up period had ended. Sensitivity analyses using clozapine to define treatment resistance (no clozapine (responder) *N* = 562; clozapine (resistant) N = 56), a definition less subject to misclassification, again found no significant associations in unadjusted and adjusted models. However given the reduced small sample size, with current small effects already identified in the overall sample (supplementary material: Fig. S.1), this analysis will only have been only powered to detect large effects (supplementary material: Table S.6 and Table S.7).

Differences between cohort samples could also encompass some heterogeneity which may have blurred potential relationships. Despite controlling for the cohort where data originated from in adjusted logistic regressions, it should be noted that these datasets originated from different geographical locations, timeframes and sampling strategies (see Smart et al., 2022). This may account for the variance in proportions of treatment resistance groups (e.g. Oslo; 1 % and West London 5 %) to treatment responsive cases. In addition, only 4 of the 7 cohorts classified TRS patients using the recommendations from the TRIPP working group (Howes et al., 2017). As documented in their systematic review of the literatures (42 studies; Howes et al., 2017), there is a large variation between studies in their use of inclusion criteria for TRS, with only 5 % of their sample (2 studies) using the same criteria. While the majority of cohorts in our sample adopted the TRIPP working group criteria, this was not consistent across cohorts which may have introduced some additional heterogeneity within our sample. Future researchers investigating differences in treatment response and resistance should endeavour to utilise the recommendations from the TRIPP working group to improve the standardization, and thus comparability, of inclusion criteria for these samples. However, in terms of cognitive data collection between cohorts, heterogeneity estimates following meta-analyses did not report any significant differences (supplementary material: Table S.2).

## 5 Conclusion

Our findings suggest that first-episode patients with an antipsychotic treatment resistant illness show poorer performance in IQ/general cognition, relative to performance in the whole sample. Although replication in larger studies is required, it appears that deficits in IQ/general cognitive functioning at first episode are associated with future treatment resistance. Cognitive variables may be able to provide further insight into neurodevelopmental factors associated with treatment resistance or act as early predictors of treatment resistance, which could allow prompt identification of refractory illness and timely intervention with evidence-based treatments such as clozapine, or augmentation/add-on strategies with other antipsychotic and mood stabilising agents. Future studies should test whether measures of IQ/general cognition improve the prediction models of treatment resistance when added to known biological and clinical predictors.

## Twitter

International prospective cohort study of FEP patients observes a relationship between early deficits in performance in measures of IQ/General cognitive function and future treatment resistance.

## Funding

This work was supported by a Stratified Medicine Programme grant to J.H.M from the 10.13039/501100000265Medical Research Council (grant number MR/L011794/1 which funded the research and supported S.E.S., A.F.P., R.M.M., J.T.R.W. & J.H.M.) E.M's PhD is funded by the 10.13039/501100000265MRC-doctoral training partnership studentship in Biomedical Sciences at King's College London. J.H.M, E.K, R.M.M are part funded by the National Institute for Health Research (NIHR) Biomedical Research Centre at South London and Maudsley NHS Foundation Trust and King's College London. A.P.K. is funded by the 10.13039/501100000272NIHR Biomedical Research Centre at South London and Maudsley NHS Foundation Trust and King's College London. O.A. is further funded by an NIHR Post-Doctoral Fellowship (PDF-2018-11-ST2-020). The views expressed are those of the authors and not necessarily those of the NHS, the MRC, the NIHR or the Department of Health. E.M.J. is supported by the UCL/UCLH Biomedical Research Centre.

The AESOP (London, UK) cohort was funded by the UK Medical Research Council (Ref: G0500817). The Bologna (Italy) cohort was funded by the European Community's Seventh Framework Program under grant agreement (agreement No. HEALTH-F2-2010–241909, Project EU-GEI). The GAP (London, UK) cohort was funded by the UK National Institute of Health Research (NIHR) Specialist Biomedical Research Centre for Mental Health, South London and Maudsley NHS Mental Health Foundation Trust (SLaM) and the Institute of Psychiatry, Psychology, and Neuroscience at King's College London; Psychiatry Research Trust; Maudsley Charity Research Fund; and the European Community's Seventh Framework Program grant (agreement No. HEALTH-F2-2009-241909, Project EU-GEI). The Oslo (Norway) cohort was funded by the Stiftelsen KG Jebsen, Research Council of Norway (#223273, under the Centers of Excellence funding scheme, and #300309, #283798) and the South-Eastern Norway Regional Health Authority (#2006233, #2006258, #2011085, #2014102, #2015088, #2017-112). The Paris (France) cohort was funded by European Community's Seventh Framework Program grant (agreement No. HEALTH-F2-2010–241909, Project EU-GEI). The Santander (Spain) cohort was funded by the following grants (to B.C.F): Instituto de Salud Carlos III, FIS 00/3095, PI020499, PI050427, PI060507, Plan Nacional de Drogas Research Grant 2005-Orden sco/3246/2004, and SENY Fundatio Research Grant CI 2005-0308007, Fundacion Marques de Valdecilla A/02/07 and API07/011. SAF2016-76046-R and SAF2013-46292-R (MINECO and FEDER). The West London (UK) cohort was funded The Wellcome Trust (Grant Numbers: 042025; 052247; 064607).

## CRediT authorship contribution statement

J.H.M., S.E.S., R.M.M., J.T.R.W., O.D.H., A.F.P., D.B., B.C.F., M.D.F., G.A.D., E.J., I.M., A.S., I.T. and A.T contributed to the design and implementation of the study. S.E.S., A.F., B.P., A.F.P., O.A.A., T.R.E.B., J.R.M., G.D., O.A., A.D., O.L., L.G., C.S and J.V.B. aided in the collection and preparation of the dataset. E.M. completed analyses and wrote the manuscript. J.H.M., O.A., E.K., T.R.E.B., S.E.S., A.P.K., I.M., I.T., A.D., E.J., O.A and G.A.D provided comments on the manuscript.

## Declaration of competing interest

J.T.R.W. is an investigator on a grant from Takeda Pharmaceuticals Ltd. to Cardiff University, for a project unrelated to the work presented here. S.E.S. is employed on this grant. M.D.F. has received a fee for educational seminars from Lundbeck and Janssen. O.A.A. is a consultant to HealthLytix and has received speakers honorarium from Lundbeck and Sunovion. T.R.E.B. has been a member of an advisory board for Gedeon Richter. B.C.F. has received honoraria for participation as a consultant and/or as a speaker at educational events from ADAMED, Mylan, Angelini, Janssen Johnson & Johnson, Lundbeck, and Otsuka Pharmaceuticals. R.M.M. has received payments for non-promotional lectures from Janssen, Otsuka, Sunovian, and Lundbeck. J.H.M. has received research funding from H Lundbeck.

## Appendix A Supplementary data

Supplementary figures Image 1

Supplementary tables Image 2

Appendix A: Descriptions of covariates and all potential auxiliary variables. Image 3
