# Corrigendum to “Cognitive performance at first episode of psychosis and the relationship with future treatment resistance: Evidence from an international prospective cohort study” [Schizophr. Res. volume 225 (May 2023) 173–181]

**DOI:** 10.1016/j.schres.2024.06.029

**Published:** 2024-08

**Authors:** Edward Millgate, Sophie E. Smart, Antonio F. Pardiñas, Eugenia Kravariti, Olesya Ajnakina, Adrianna P. Kępińska, Ole A. Andreassen, Thomas R.E. Barnes, Domenico Berardi, Benedicto Crespo-Facorro, Giuseppe D'Andrea, Arsime Demjaha, Marta Di Forti, Gillian A. Doody, Alp Üçok, Laura Kassoumeri, Aziz Ferchiou, Lorenzo Guidi, Eileen M. Joyce, Ornella Lastrina, Ingrid Melle, Baptiste Pignon, Jean-Romain Richard, Carmen Simonsen, Andrei Szöke, Ilaria Tarricone, Andrea Tortelli, Javier Vázquez-Bourgon, Robin M. Murray, James T.R. Walters, James H. MacCabe, STRATA Consortium

**Affiliations:** a Department of Psychosis Studies, Institute of Psychiatry, Psychology & Neuroscience, King's College London, London, UK; b MRC Centre for Neuropsychiatric Genetics and Genomics, Division of Psychological Medicine and Clinical Neurosciences, Cardiff University, Cardiff, UK; c Department of Biostatistics & Health Informatics, Institute of Psychiatry, Psychology & Neuroscience, King's College London, London, UK; d Department of Behavioural Science and Health, Institute of Epidemiology and Health Care, University College London, London, UK; e Norwegian Centre for Mental Disorders Research (NORMENT), Institute of Clinical Medicine, University of Oslo, Oslo, Norway; f Division of Mental Health and Addiction, Oslo University Hospital, Oslo, Norway; g Division of Psychiatry, Imperial College London, UK; h Department of Biomedical and Neuro-motor Sciences, Psychiatry Unit, Alma Mater Studiorum Università di Bologna, Bologna, Italy; i Department of Psychiatry, Hospital Universitario Virgen del Rocio, IBiS, Universidad de Sevilla, Spain; j Centro de Investigacion en Red Salud Mental (CIBERSAM), Sevilla, Spain; k Social Genetics and Developmental Psychiatry, Institute of Psychiatry, Psychology & Neuroscience, King's College London, London, UK; l South London and Maudsley NHS Mental Health Foundation Trust, London, UK; m Department of Medical Education, University of Nottingham Faculty of Medicine and Health Sciences, Nottingham, UK; n Univ Paris Est Creteil, INSERM, IMRB, Translational Neuropsychiatry, Fondation FondaMental, Creteil, France; o AP-HP, Hôpitaux Universitaires H. Mondor, DMU IMPACT, FHU ADAPT, Creteil, France; p Department of Medical and Surgical Sciences, Bologna Transcultural Psychosomatic Team (BoTPT), Alma Mater Studiorum – University of Bologna, Bologna, Italy; q Department of Clinical and Movement Neuroscience, UCL Queen Square Institute of Neurology, University College London, London, UK; r Early Intervention in Psychosis Advisory Unit for South East Norway (TIPS Sør-Øst), Division of Mental Health and Addiction, Oslo University Hospital, Norway; s Groupe Hospitalier Universitaire Psychiatrie Neurosciences Paris, Pôle Psychiatrie Précarité, Paris, France; t Department of Psychiatry, University Hospital Marques de Valdecilla - Instituto de Investigación Marques de Valdecilla (IDIVAL), Santander, Spain; u Department of Medicine and Psychiatry, School of Medicine, University of Cantabria, Santander, Spain; v Istanbul University, Istanbul Faculty of Medicine, Department of Psychiatry, Istanbul, Turkey

i. The authors regret to inform that during the peer-review process (2nd revision) an author who had provided data which was later included during revisions was not included in the final author list. As we provide authorship to all data contributors please find the details of the additional author below highlighted in red, with his affiliation to be added below:

Edward Millgate, Sophie E Smart, Antonio F Pardiñas, Eugenia Kravariti, Olesya Ajnakina, Adrianna P Kępińska, Ole A Andreassen, Thomas RE Barnes, Domenico Berardi, Benedicto Crespo-Facorro, Giuseppe D'Andrea, Arsime Demjaha, Marta Di Forti, Gillian A Doody, , Laura Kassoumeri, Aziz Ferchiou, Lorenzo Guidi, Eileen M Joyce, Ornella Lastrina, Ingrid Melle, Baptiste Pignon, Jean-Romain Richard, Carmen Simonsen, Andrei Szöke, Ilaria Tarricone, Andrea Tortelli, Javier Vázquez-Bourgon, Robin M Murray, James TR Walters, James H MacCabe, STRATA Consortium.

With his corresponding affiliation as:

**Figure:**
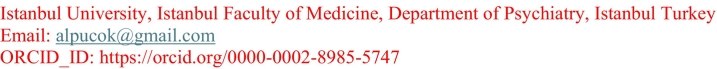

ii. We have noticed some unecessary information within Table 1: the number or errors and time taken in seconds are already in the Trail Making Test B section for Executive Function, so these can just be removed from the CANTAB section and don't need to be added in elsewhere. So please can these variables be removed accordingly from Table 1.

The authors would like to apologise for any inconvenience caused.

