# Supplementary material for: Cognitive performance at first episode of psychosis and the relationship with future treatment resistance: Evidence from an international prospective cohort study
Source: Schizophr Res. 2023 May;255:173–81. doi: 10.1016/j.schres.2023.03.020 (PMC10390338; doi:10.1016/j.schres.2023.03.020)
Supplement: Supplementary file 1 — Supplementary figures [file mmc1.pdf]

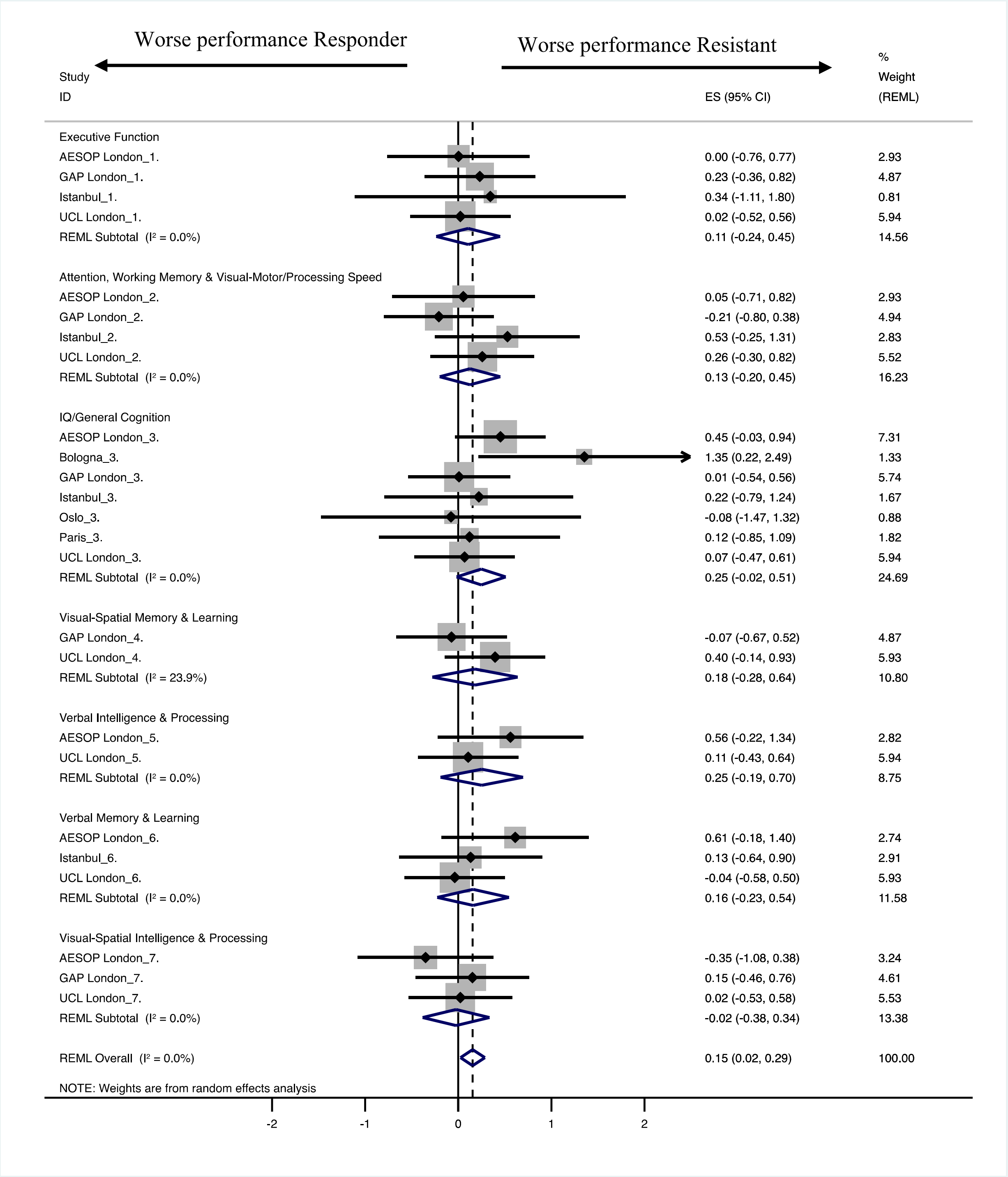

Figure S.1. Forest plots of effect sizes of baseline cognitive performance differences between antipsychotic responders (Responders) and antipsychotic resistant (Resistant) samples in 1. Executive Function, 2. Attention, Working Memory & Visual-Motor/Processing Speed, 3. IQ/General Cognition, 4. Visual-Spatial Memory & Learning, 5. Verbal Intelligence & Processing, 6. Verbal Memory & Learning, 7. Visual-Spatial Intelligence.

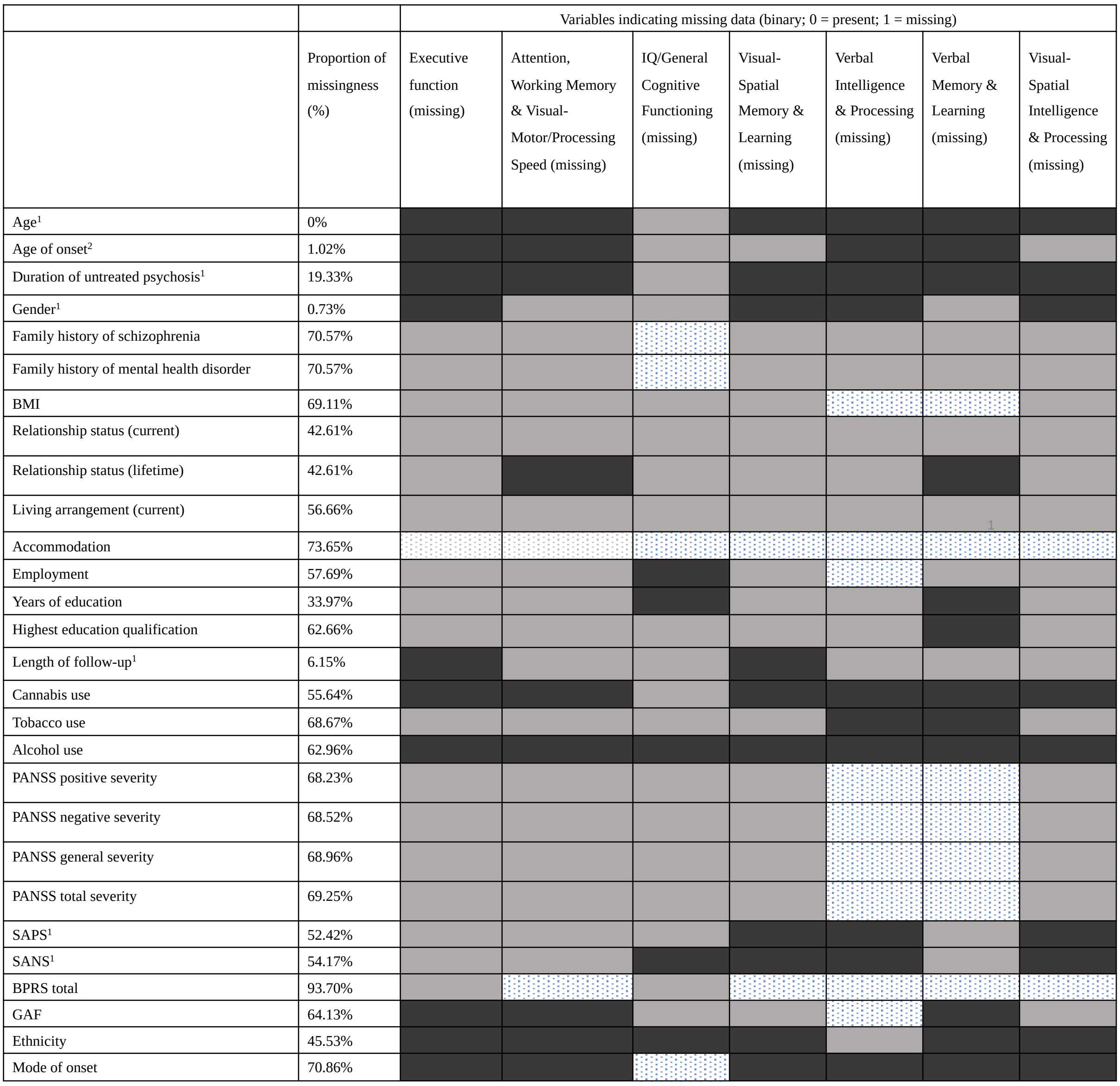

		Variables indicating missing data (binary; 0 = present; 1 = missing)						
	Proportion of missingness (%)	Executive function (missing)	Attention, Working Memory & Visual-Motor/Processing Speed (missing)	IQ/General Cognitive Functioning (missing)	Visual-Spatial Memory & Learning (missing)	Verbal Intelligence & Processing (missing)	Verbal Memory & Learning (missing)	Visual-Spatial Intelligence & Processing (missing)
Age[1]	0%							
Age of onset[2]	1.02%							
Duration of untreated psychosis[1]	19.33%							
Gender[1]	0.73%							
Family history of schizophrenia	70.57%							
Family history of mental health disorder	70.57%							
BMI	69.11%							
Relationship status (current)	42.61%							
Relationship status (lifetime)	42.61%							
Living arrangement (current)	56.66%							
Accommodation	73.65%							
Employment	57.69%							
Years of education	33.97%							
Highest education qualification	62.66%							
Length of follow-up[1]	6.15%							
Cannabis use	55.64%							
Tobacco use	68.67%							
Alcohol use	62.96%							
PANSS positive severity	68.23%							
PANSS negative severity	68.52%							
PANSS general severity	68.96%							
PANSS total severity	69.25%							
SAPS[1]	52.42%							
SANS[1]	54.17%							
BPRS total	93.70%							
GAF	64.13%							
Ethnicity	45.53%							
Mode of onset	70.86%							

Figure S.2. Association plot between clinical and demographic variables and the proportion of missingness for each cognitive domain.

Note. [1] = included as covariate in analyses, [2] = also included as auxiliary variable. Dark grey = association/effect found; light grey = no association/effect found, dots = analysis not executed

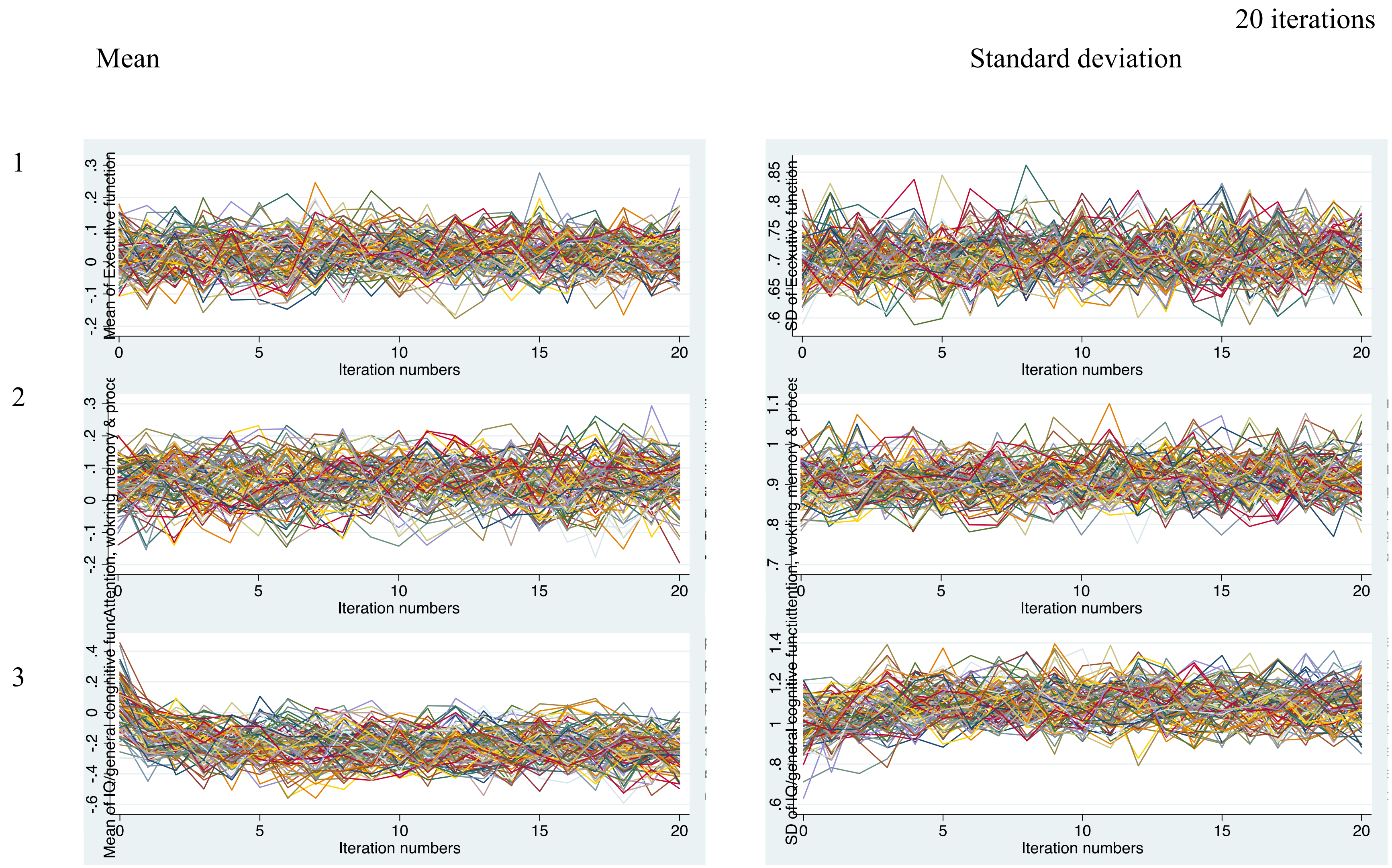

Figure S.3.1 Convergence plots for multiple imputations by chained equations (MICE) at 20 iterations and 100 imputations. Means and standard deviations are presented for 1. executive function, 2. attention, working memory and processing speed and 3. IQ/general cognitive functioning.

20 iterations

Mean | Standard deviation

4

5

6

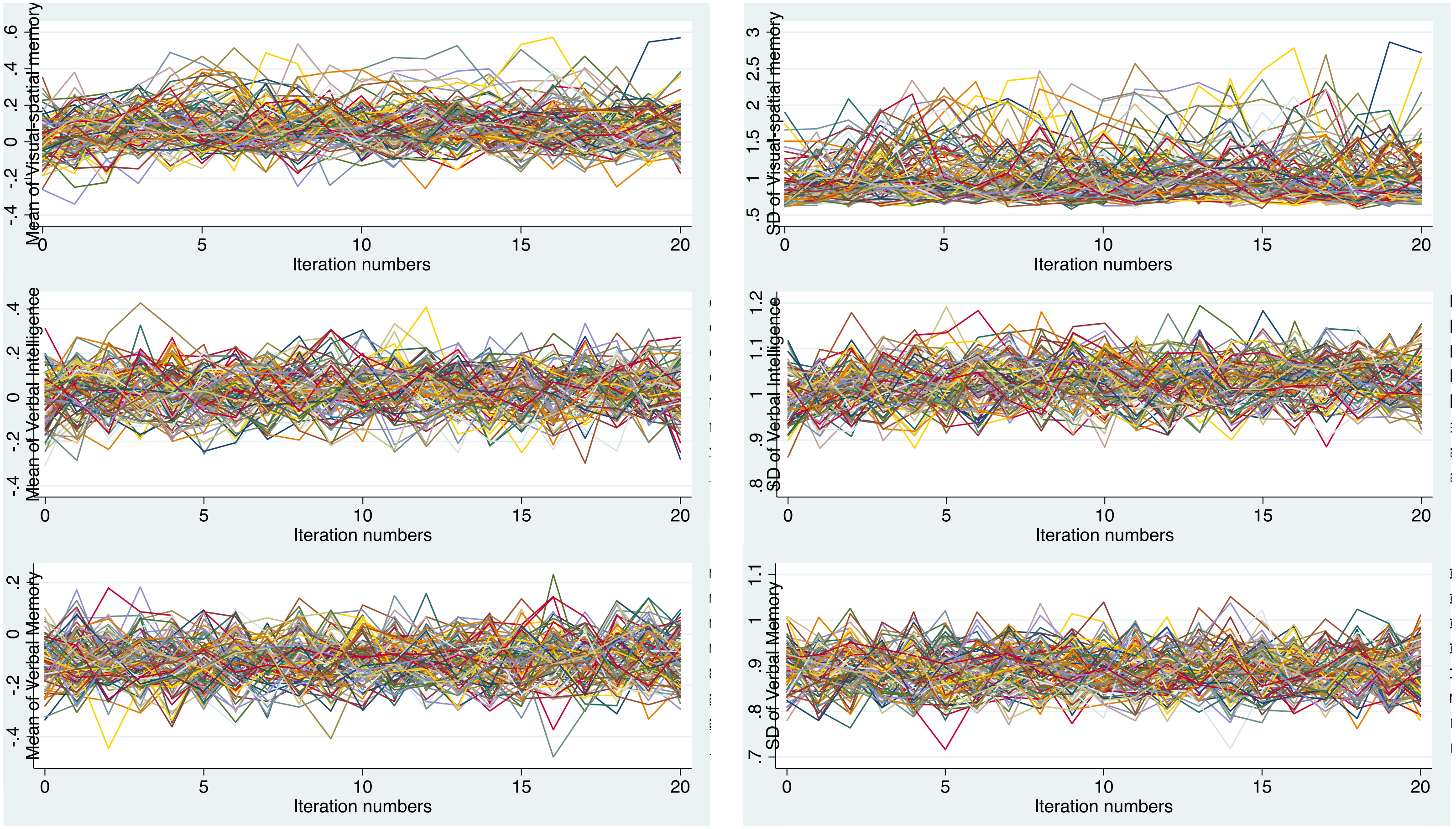

Figure S.3.2 Convergence plots for multiple imputations by chained equations (MICE) at 20 iterations and 100 imputations. Means and standard deviations are presented for 3. visual-spatial memory and learning, 4. verbal intelligence and processing and 5. verbal memory and learning.

20 iterations

Mean

Standard deviation

7

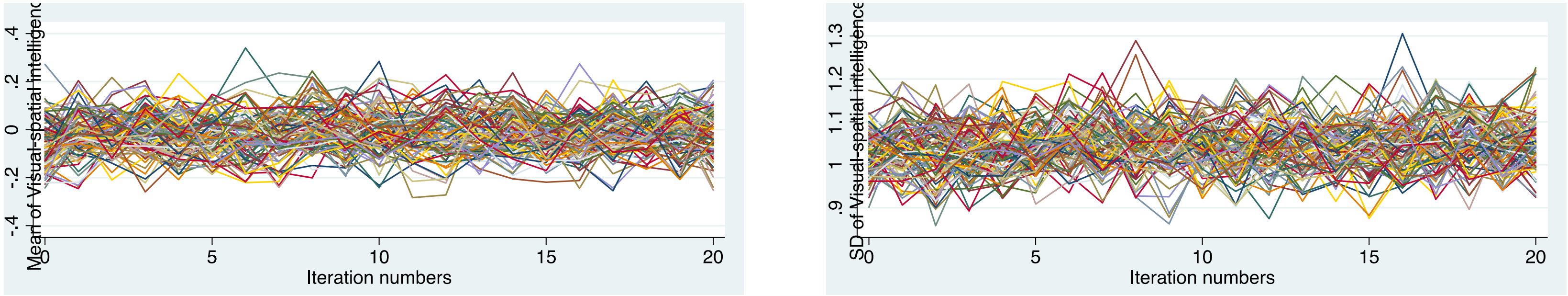

Figure S.3.3 Convergence plots for multiple imputations by chained equations (MICE) at 20 iterations and 100 imputations. Means and standard deviations are presented for 7. visual-spatial intelligence and processing.

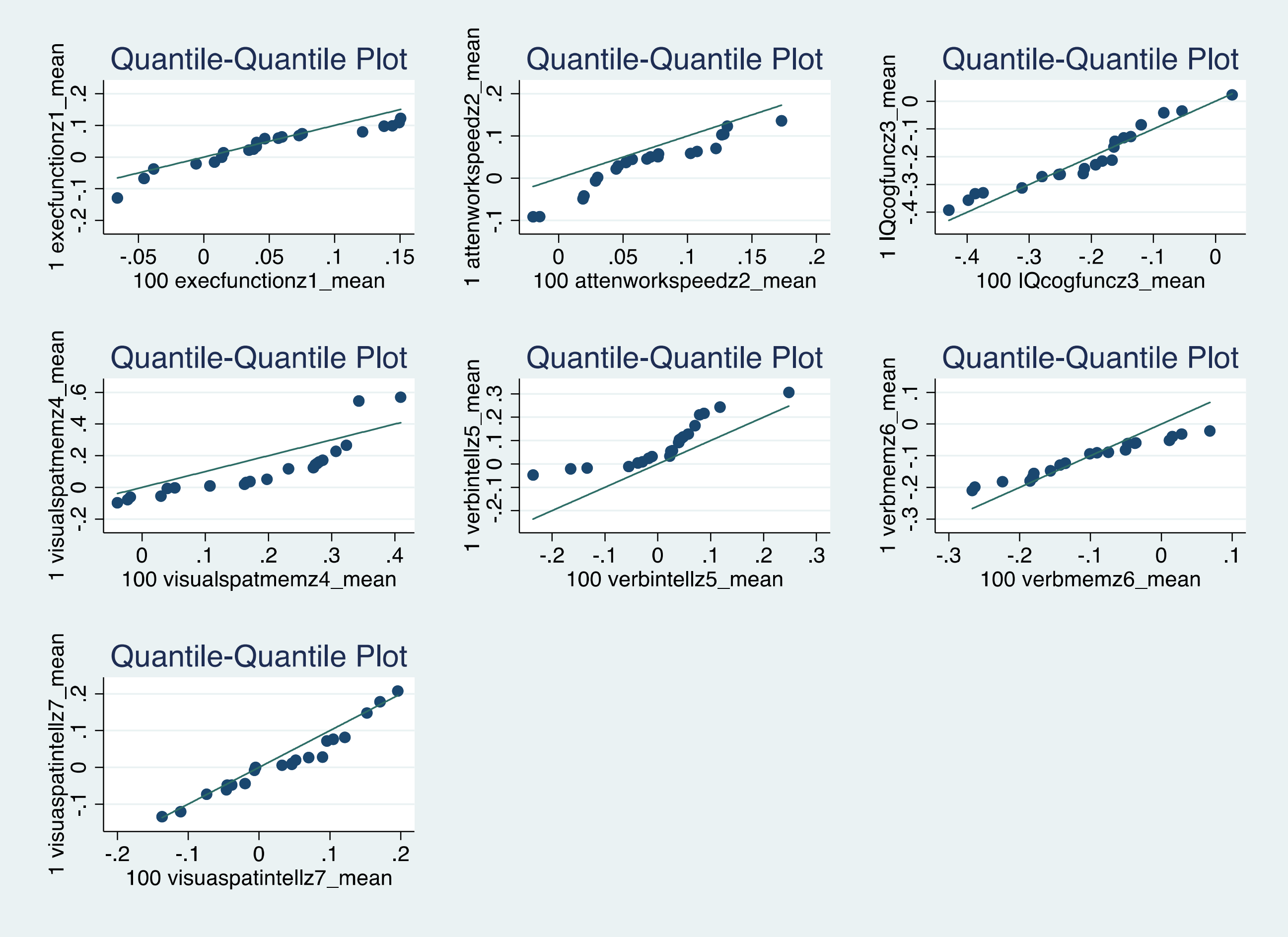

Figure S.4 Quantile-quantile plots of original mean values (dotted) against imputed values for the 100th imputation for each cognitive domain.
