# Supplementary material for: Cognitive performance at first episode of psychosis and the relationship with future treatment resistance: Evidence from an international prospective cohort study
Source: Schizophr Res. 2023 May;255:173–81. doi: 10.1016/j.schres.2023.03.020 (PMC10390338; doi:10.1016/j.schres.2023.03.020)
Supplement: Supplementary file 2 — Supplementary tables [file mmc2.docx]

**Supplementary material**

**Table S.1. Definitions for treatment resistance and treatment response per each cohort**

| Cohort | Criteria for treatment resistance | Criteria for treatment response |
| --- | --- | --- |
| AESOP (London, Nottingham, UK) | Treated with clozapine during the follow-up period.  OR  Two sequential antipsychotic trials: each of at least 4 weeks’ duration at a daily dose of 400–600 mg of chlorpromazine equivalents.  Persistent psychotic symptoms: a rating of at least moderate severity on one or more positive symptoms as rated by SCAN (WHO, 1994).  Recorded adherence to medication.  In line with NICE criteria (National Institute for Health and Care Excellence (NICE), 2014). | Treatment response: a state, of at least 6 months’ duration, in which no symptoms or only symptoms of mild severity, not interfering with daily functioning, were experienced (Andreasen *et al.*, 2005) |
| EUGEI & BoFEP (Bologna, Italy) | Treated with or considered for clozapine during the follow-up period.  OR  Two or more antipsychotic trials: at least two different antipsychotics, each of at least 6 weeks’ duration, with dosages in, at least, the mid-point of the licensed therapeutic range.  Moderate psychotic symptoms: a rating of at least 70 on the PANSS.  Moderate functioning: at least moderate functional impairment measured using the Clinical Global Impression (CGI) scale (Busner and Targum, 2007).  In line with Treatment Response and Resistance in Psychosis working group (TRRIP) criteria (Howes *et al.*, 2017). | Never treated with or considered for clozapine.  AND  Does not meet criteria for treatment resistance. |
| GAP (London, UK) | Treated with or considered for clozapine during the follow-up period.  OR  Two sequential antipsychotic trials: each of at least 6 weeks’ duration at a daily dose of 400 mg of chlorpromazine equivalents (Leucht *et al.*, 2015).  Persistent psychotic symptoms: Little or no symptomatic improvement.  Excluded those who were intolerant of antipsychotic medications or those who self-discontinued medication. | Never treated with or considered for clozapine.  AND  Does not meet criteria for treatment resistance. |
| Istanbul (Turkey) | Treated with or considered for clozapine during the follow up period.  OR  Two or more antipsychotic trials: at least two different antipsychotics, each of at least 6 weeks’ duration, with, at least, medium dosages of the dose range.  Persistent psychotic symptoms: comparison of current and previous BPRS scores, alongside opinion of family member and treating psychiatrist. | Never treated with or considered for clozapine.  AND  Does not meet criteria for treatment resistance. |
| TOP (Oslo, Norway) | Treated with or considered for clozapine during the follow-up period.  OR  Two or more antipsychotic trials: at least two different antipsychotics, each of at least 6 weeks’ duration, (no minimum dose).  Moderate psychotic symptoms: a rating of at least moderate severity as rated by the PANSS (minimum of 4 on at least 2 positive-subscale items (or a minimum of 6 on 1 positive-subscale item) for at least 12 weeks.  Moderate functioning: at least moderate functional impairment measured using the GAF scale (score less than or equal to 51).  In line with Treatment Response and Resistance in Psychosis working group (TRRIP) criteria (Howes *et al.*, 2017). | Never treated with or considered for clozapine.  AND  Does not meet criteria for treatment resistance. |
| Paris (France) | Treated with or considered for clozapine during the follow-up period.  OR  Two or more antipsychotic trials: at least two different antipsychotics, each of at least 6 weeks’ duration, with dosages in at least the mid-point of the licensed therapeutic range.  Moderate psychotic symptoms: a rating of at least moderate severity, as rated by the PANSS, for at least 12 weeks.  Moderate functioning: at least moderate functional impairment measured using the GAF scale.  In line with Treatment Response and Resistance in Psychosis working group (TRRIP) criteria (Howes *et al.*, 2017) | Never treated with or considered for clozapine.  AND  Does not meet criteria for treatment resistance. |
| West London (London, UK) | Treated with clozapine at the time of a follow-up interview. | Never treated with clozapine at the time of a follow-up interview. |

*Note. AESOP* = Aetiology and Ethnicity in Schizophrenia and Other Psychoses study; *EUGEI* = European Network of National Schizophrenia Networks Studying Gene-Environment Interactions study; *BoFEP* = Bologna first episode psychosis study; *GAP* = Genetics and Psychosis study; *TOP* = Thematic Organized Psychosis Research study; *TRRIP* = Treatment Response and Resistance in Psychosis working group; *SCAN* = Schedules for Clinical Assessment in Neuropsychiatry; *WHO* = World Health Organisation; *NICE* = National Institute for Health and Care Excellence; *PANSS* = Positive and Negative Syndrome Scale; *BPRS* = Brief Psychiatric Rating Scle; *CGI* = Clinical Global Impression; *GAF* = Global Assessment of Functioning.

**Table S.2 Meta-analyses of performance differences between baseline cognitive outcomes (standardised to the whole sample) between treatment resistant and treatment responsive samples**

| Cognitive domain | Effect size (*reml*) | L95CI% | U95CI% | Cochrane Q |
| --- | --- | --- | --- | --- |
| Executive Function | 0.11 | -0.24 | 0.45 | 0.43, *p* = .933 |
| Attention, Working Memory & Visual-Motor/Processing Speed | 0.13 | -0.20 | 0.45 | 2.51, *p* = .473 |
| IQ/General Cognition | 0.25 | -0.02 | 0.51 | 5.77, *p* = .450 |
| Visual-Spatial Memory & Learning | 0.18 | -0.28 | 0.64 | 1.31, *p* = .252 |
| Verbal Intelligence & Processing | 0.25 | -0.20 | 0.70 | 0.88, *p* = .348 |
| Verbal Memory & Learning | 0.16 | -0.23 | 0.54 | 1.77, *p* = .413 |
| Visual-Spatial Intelligence | -0.02 | -0.38 | 0.34 | 1.12, *p* = .571 |

*Note.* A positive effect size denotes worse performance in treatment resistant samples.

**Table S.3 Summary statistics of cognitive domains (standardised to the whole sample mean and SD) at first episode prior to MICE imputations (N = 683)**

|  |  | Treatment responsive | |  | Treatment resistant | |  | |  |
| --- | --- | --- | --- | --- | --- | --- | --- | --- | --- |
| Cognitive domain | % Missing | N | Mean | SD | N | Mean | | SD | |
| Executive function | 44.36 | 340 | 0.03 | 0.65 | 40 | -0.03 | | 0.63 | |
| Attention, Working Memory & Visual-Motor/Processing Speed | 41.29 | 356 | 0.003 | 0.87 | 45 | -0.06 | | 0.71 | |
| IQ/General Cognitive Functioning | 8.64 | 553 | 0.07 | 1.01 | 71 | -0.30 | | 1.00 | |
| Visual-Spatial Memory & Learning | 50.22 | 312 | -0.01 | 0.59 | 28 | -0.11 | | 0.75 | |
| Verbal Intelligence & Processing | 56.95 | 270 | .010 | 0.96 | 24 | -0.26 | | 0.98 | |
| Verbal Memory & Learning | 51.83 | 297 | -0.002 | 0.86 | 32 | -0.19 | | 0.70 | |
| Visual-Spatial Intelligence & Processing | 48.02 | 317 | -0.03 | 1.00 | 38 | -0.05 | | 0.89 | |

**Table S.4 Summary statistics of cognitive domains at first episode prior to MICE imputations restricted to individuals with a schizophrenia diagnosis (antipsychotic responder N = 358, antipsychotic resistant N = 57)**

|  |  | Treatment responsive schizophrenia | |  | Treatment resistant schizophrenia | |  | |  |
| --- | --- | --- | --- | --- | --- | --- | --- | --- | --- |
| Cognitive domain | % Missing | N | Mean | SD | N | Mean | | SD | |
| Executive function | 39.28% | 223 | -0.04 | 0.65 | 29 | 0.05 | | 0.57 | |
| Attention, Working Memory & Visual-Motor/Processing Speed | 38.55% | 222 | -0.42 | 0.90 | 33 | 0.09 | | 0.70 | |
| IQ/General Cognitive Functioning | 9.16% | 324 | 0.05 | 1.00 | 53 | -0.20 | | 1.02 | |
| Visual-Spatial Memory & Learning | 46.02% | 204 | 0.06 | 0.56 | 20 | 0.08 | | 0.65 | |
| Verbal Intelligence & Processing | 55.18% | 170 | -0.12 | 0.92 | 16 | -0.09 | | 0.99 | |
| Verbal Memory & Learning | 52.29% | 177 | .002 | 0.88 | 21 | -0.10 | | 0.69 | |
| Visual-Spatial Intelligence & Processing | 43.37% | 206 | -0.041 | 0.97 | 29 | 0.08 | | 0.80 | |

**Table S.5**

*Logistic regression models for unadjusted and adjusted models comparing the relationship between baseline cognitive performance and treatment resistance restricted to those with a diagnosis for schizophrenia*

|  | Unadjusted | | | | | Adjusted | | | | |
| --- | --- | --- | --- | --- | --- | --- | --- | --- | --- | --- |
| Domain | β | SE | 95%CI | OR | P-value | β | SE | 95%CI | OR | P-value |
| Executive function | 0.07 | 0.30 | -0.51 ; 0.66 | 1.08 | .804 | 0.01 | 0.32 | -0.63 ; 0.64 | 1.01 | .989 |
| Attention, Working Memory & Visual-Motor/Processing Speed | 0.03 | 0.20 | -0.36 ; 0.41 | 1.03 | .890 | 0.02 | 0.24 | -0.46 ; 0.50 | 1.02 | .948 |
| IQ/General Cognition | -0.20 | 0.14 | -0.49 ; 0.08 | 0.82 | .155 | -0.17 | 0.17 | -0.50 ; 0.17 | 0.85 | .322 |
| Visual-Spatial Memory & Learning | 0.16 | 0.40 | -0.63 ; 0.94 | 1.17 | .693 | -0.16 | 0.46 | -1.05 ; 0.74 | 0.86 | .733 |
| Verbal Intelligence & Processing | -0.27 | 0.21 | -0.67 ; 0.14 | 0.77 | .199 | -0.20 | 0.27 | -0.72 ; 0.33 | 0.82 | .464 |
| Verbal Memory & Learning | -0.08 | 0.23 | -0.54 ; 0.38 | 0.92 | .722 | 0.01 | 0.28 | -0.53 ; 0.55 | 1.01 | .964 |
| Visual-Spatial Intelligence & Processing | 0.04 | 0.19 | -0.34 ; 0.42 | 1.04 | .838 | 0.13 | 0.26 | -0.39 ; 0.64 | 1.13 | .634 |

Adjusted: adjusted for age, gender, cohort, duration of untreated psychosis, length of follow-up, SAPS & SANS

*Note.* OR = Odds Ratio; CIs = confidence intervals

**Table S.6 Summary statistics of cognitive domains at first episode prior to MICE imputations comparing treatment resistant and treatment responsive individuals where clozapine use was used to define treatment resistance (No clozapine N = 562, Clozapine N = 56)**

|  |  | No Clozapine | |  | Clozapine use | |  | |  |
| --- | --- | --- | --- | --- | --- | --- | --- | --- | --- |
| Cognitive domain | % Missing | N | Mean | SD | N | Mean | | SD | |
| Executive function | 42.56% | 323 | 0.03 | 0.63 | 32 | -0.06 | | 0.67 | |
| Attention, Working Memory & Visual-Motor/Processing Speed | 39.16% | 339 | -0.003 | 0.87 | 37 | -0.03 | | 0.72 | |
| IQ/General Cognitive Functioning | 9.39% | 510 | 0.05 | 1.01 | 50 | -0.19 | | 0.98 | |
| Visual-Spatial Memory & Learning | 45.79% | 312 | -0.01 | 0.59 | 23 | -0.21 | | 0.72 | |
| Verbal Intelligence & Processing | 55.66% | 253 | -0.001 | 0.95 | 21 | -0.15 | | 1.00 | |
| Verbal Memory & Learning | 49.84% | 281 | -0.006 | 0.85 | 29 | -0.15 | | 0.72 | |
| Visual-Spatial Intelligence & Processing | 46.44% | 301 | -0.02 | 0.99 | 30 | 0.12 | | 0.87 | |

**Table S.7**

*Logistic regression models for unadjusted and adjusted models comparing the relationship between baseline cognitive performance and clozapine use*

|  | Unadjusted | | | | | Adjusted | | | | |
| --- | --- | --- | --- | --- | --- | --- | --- | --- | --- | --- |
| Domain | β | SE | 95%CI | OR | P-value | β | SE | 95%CI | OR | P-value |
| Executive function | -0.26 | 0.28 | -0.80 ; 0.28 | 0.77 | .348 | -0.16 | 0.31 | -0.76 ; 0.44 | 0.85 | .596 |
| Attention, Working Memory & Visual-Motor/Processing Speed | -0.12 | 0.89 | -0.48 ; 0.25 | 0.54 | .535 | -0.06 | 0.21 | -0.48 ; 0.36 | 0.94 | .781 |
| IQ/General Cognition | -0.23 | 0.14 | -0.50 ; 0.05 | 0.80 | .106 | -0.11 | 0.17 | -0.44 ; 0.22 | 0.90 | .518 |
| Visual-Spatial Memory & Learning | -0.38 | 0.40 | -1.01 ; 0.25 | 0.68 | .235 | -0.61 | 0.32 | -1.24 ; 0.01 | 0.54 | .055 |
| Verbal Intelligence & Processing | -0.23 | 0.19 | -0.59 ; 0.14 | 0.80 | .226 | -0.06 | 0.23 | -0.50 ; 0.39 | 0.95 | .804 |
| Verbal Memory & Learning | -0.24 | 0.22 | -0.68 ; 0.20 | 0.79 | .279 | -0.12 | 0.25 | -0.61 ; 0.37 | 0.89 | .629 |
| Visual-Spatial Intelligence & Processing | 0.01 | 0.18 | -0.35 ; 0.38 | 1.01 | .940 | 0.10 | 0.22 | -0.33 ; 0.53 | 1.11 | .648 |

Adjusted: adjusted for age, gender, cohort, duration of untreated psychosis, length of follow-up, SAPS & SANS

*Note.* OR = Odds Ratio; CIs = confidence interval.
