# Supplementary material for: Cognitive performance at first episode of psychosis and the relationship with future treatment resistance: Evidence from an international prospective cohort study
Source: Schizophr Res. 2023 May;255:173–81. doi: 10.1016/j.schres.2023.03.020 (PMC10390338; doi:10.1016/j.schres.2023.03.020)
Supplement: Supplementary file 3 — Appendix A: Descriptions of covariates and all potential auxiliary variables. [file mmc3.docx]

**Appendix A: Descriptions of covariates and all potential auxiliary variables**

**Covariates:**

*Age at baseline*

Participant’s age at the time of the baseline assessment was available for all samples.

*Gender*

For all cohorts, gender was a binary variable and participants were either categorised as male or female.

*Duration of untreated psychosis (DUP)*

DUP is the time in days between the first occurrence of psychotic symptoms and the start of antipsychotic treatment for psychosis. DUP was recorded in AESOP, Oslo, Paris, Santander, and West London. DUP measured in weeks (Oslo, Paris, and West London) was converted to days by multiplying by seven. DUP measured in months (West London) was converted to days by multiplying by 30.417.

*Scale for the Assessment of Negative Symptoms (SANS)*

SANS scores were recorded in Santander and West London. The SANS is used to rate negative symptoms in the following five domains: flat affect, alogia, apathy, anhedonia, and attention (Andreasen, 1983; Andreasen, 1989). Within each domain there are a number of individual items and one global item. Each item is rated on a 0-5 Likert scale (0 equates ‘absent’ and 5 equates to ‘severe’). The SANS composite total is a sum of all SANS items apart from the global items (items: 1- 7, 9-12, 14-16, 18-21, and 23-24) the minimum score is 0 and the maximum score is 100. The SANS global summary score is a sum of all the five global items (items: 8, 13, 17, 22, 25), the minimum score is 0 and the maximum score is 25. SANS global summary scores were recorded in Santander and West London. The SANS item ‘inappropriate affect’ is sometimes dropped from the composite total score because it does not correlate with the overall subscale score (Andreasen, 1982). This item was not recorded in West London. As individual items were not available, SANS global summary scores were used.

*Scale for the Assessment of Positive Symptoms (SAPS)*

SAPS scores were recorded in Santander and West London. The SAPS is used to rate positive symptoms in the following four domains: hallucinations, delusions, bizarre behaviour, and thought disorder (Andreasen, 1984). Within each domain there are a number of individual items and one global item. Each item is rated on a 0-5 Likert scale (0 equates ‘absent’ and 5 equates to ‘severe’). The SAPS composite total is a sum of all SAPS items apart from the global items (items: 1-6, 8-19, 21-24, and 26-33). The SAPS global summary score is a sum of all the five global items (items: 7, 20, 25, 34). SANS composite total and global summary scores were recorded in Santander and West London. In line with the SANS scores available, only SANS global summary scores were used.

**Auxiliary variables:**

*Employment*

Employment circumstances at baseline were recorded in Bologna, GAP, Oslo, and Paris, however, each used a different ordinal scale. From this information a binary variable was created: employed vs. unemployed. I considered ‘unemployed’ to include retired, economically inactive, rehabilitation welfare, disability benefit, and sick leave. Students were considered as ‘employed’ because in Oslo studying and working were grouped together.

*Ethnicity*

Ethnicity was recorded at baseline for all cohorts. Three categories were used to define ethnicity: European, (e.g. White British, White other, White UK, White Irish, White Italian, White East European, Caucasian, European, American, and Gipsy), Black (e.g. Black Caribbean, Black African, Maghreb, African, Black), and Asian/Mixed/Other (e.g. Asian, Mixed Black, Mixed Other, Other, White and Black African, Arab, Filipino, Chinese, Bangladeshi, Indian, Middle East, Hispanic, and Latin American).

*Alcohol*

Alcohol use at baseline was recorded in Santander, Oslo and West London, however each used a different scale. From this information, I created a binary variable: non-drinker vs. drinker. Alcohol use (yes vs. no) was recorded at baseline in Santander. Alcohol use in the last six months, measured in number of units, was recorded in Oslo. Zero units in the last six months was classified as ‘non-drinker’, while more than zero units in the last six months was classified as ‘drinker’. Alcohol use was also recorded in West London (three categories: no, yes, and dependent). No was classified as ‘non-drinker’, while yes and dependent were classified as ‘drinker’.

*Body Mass Index (BMI)*

BMI at baseline was recorded in Oslo and Santander. Weight in kilograms (kg) and height in centimetres (cm) at baseline was recorded in Belfast and GAP. The following formula was used to calculate BMI in Belfast and GAP: (weight/(height/100))/(weigh/100).

*Cannabis*

Cannabis use at baseline was recorded in Bologna, GAP, Oslo, Santander and West London, however each used a different scale. From this information a binary variable was created: cannabis vs. no cannabis. Cannabis use (yes vs. no) was recorded at baseline in Bologna, GAP, Santander, and West London. Cannabis use in the last six months (yes vs. no) was recorded in Oslo. No use in the last six months was classified as ‘no cannabis’, while use in the last six months was classified as ‘cannabis’.

*Education qualifications*

Highest educational qualification was recorded at baseline for AESOP, Bologna, GAP and Paris. Four categories were used to define highest educational qualification: None (e.g. no qualifications, primary school, school without qualification), Basic (e.g. GCSE, school with qualifications, O levels, junior/high school, secondary school), Further (e.g. first level on non- compulsory education, A levels, high school, Baccalaureate, vocation or college BTEC, NVQ, technical college), and Higher (university, undergraduate degree, postgraduate degree, professional).

*Living*

Living circumstances at baseline were recorded in AESOP, Bologna, GAP, Oslo and Santander, however, each used a different ordinal scale to capture this data. Three binary variables were created: living with company vs living alone (e.g. spouse, children, family, parents, friends, other vs. alone), living with family vs. with non-family (e.g. partner, children, parents, other family vs. shared housing, friends), and living with parents vs. living with non-parents (e.g. parents vs. partner, children, other family, friends).

For living with company vs. living alone, a variable from Santander which grouped living alone without children and living alone with children together was categorised as living alone. For living with family vs. with non-family, the categories ‘alone’ and ‘other’ were not used. For living with parents vs. living with non-parents, data from Oslo was not used as they grouped parents and other family members into the same category.

*Relationship*

Relationship status at baseline were recorded in AESOP, Bologna, GAP, Oslo, Paris and Santander, however, each used a different ordinal scale to capture this data. From this information, two binary variables were constructed: current relationship and lifetime relationship; in a relationship vs not in a relationship (e.g. married, steady relationship, cohabiting, civil partnership vs. single, separated, divorced, widowed, never married and not cohabiting) and ever been in a relationship vs. never been in a relationship (e.g. married, steady relationship, cohabiting, civil partnership, separated, divorced, widowed vs. single, never married and not cohabiting)

*Tobacco*

Tobacco use (yes vs. no) was recorded at baseline in Bologna, GAP, Santander and West London. A binary variable, non- smoker vs. smoker, was created.

*Years in education*

Number of years in education was recorded at baseline for Bologna, GAP, Oslo, Paris and West London.

*Age at onset*

Age of onset was considered to be the participant’s age when psychotic symptoms first occurred. Age of first psychotic symptoms was recorded at baseline for AESOP, Oslo and Santander. If this variable was not available, the participant’s age when they first presented to clinical services for psychosis was used. Age of first presentation to clinical services for psychosis was recorded at baseline for GAP, and Paris. Age of first presentation to clinical services for psychosis was recorded at the five-year follow-up for Bologna and since this is considered a static trait this data was included. As date of first presentation to clinical services is likely to be systematically later than date of first psychotic symptoms, a correction to age at first presentation to clinical services was applied when it was used instead of true age of onset. One cohort included in STRATA-G, AESOP, collected data on both age at first presentation and age of first symptoms. The mean difference between these ages was 0.547 years. Therefore, the estimated age of onset, in the absence of the variable, equalled age at first presentation to clinical services minus 0.547 years.

*Brief Psychiatric Rating Scale (BPRS)*

BPRS scores were recorded in Santander. The BPRS is designed to measure psychiatric symptoms, including hallucinations, depression, anxiety, and usual behaviour, across 24 items using a 1-7 Likert scale (1 equates ‘not present’ and 7 equates to ‘extremely severe’) (Overall & Gorham, 1962). The minimum score on the BPRS is 24 while the maximum is 168.

*Family history of psychosis*

Family history of psychosis (yes vs. no) was recorded in AESOP and West London. Parental and family history of psychosis was recorded in AESOP, using the Family Interview for Genetic Studies (FIGS; Maxwell, 1992) and family history of schizophrenia was recorded in West London, using the Diagnostic Interview for Psychosis (DIP; Castle et al., 2006). This included all known family members, and not just first-degree relatives.

*Family history of mental health disorders*

Family history of mental health disorders (yes vs. no) was recorded in AESOP. Parental and family history of any mental health disorder was recorded in AESOP, using the FIGS.

*Global Assessment of Functioning (GAF)*

GAF scores were recorded in GAP, Oslo and Paris. The GAF is a scale, included in the DSM-IV, which is used to assess social, occupational, and psychological functioning (American Psychiatric Association, 2000). Individuals are given a score from 100 (extremely high functioning) to 1 (severely impaired). The GAF is often rated by focusing on symptoms (GAF-S) only or on functioning/disability (GAF-F) only; the GAF, as a single score, is the most serve of the GAF-S and GAF-F. In GAP, Oslo, and Paris, the GAF was recorded as two scores, and for each individual the most severe score was used.

*Positive and Negative Syndrome Scale (PANSS)*

PANSS scores were recorded in GAP and Oslo. The PANSS is designed to measure symptoms of schizophrenia across 30 items using a 1-7 Likert scale for each domain (1 equates to ‘absent’ and 7 to ‘extreme’) (Kay, Fiszbein, & Opler, 1987). The minimum score on the PANSS is 30 while the maximum is 210. There are three subscales within the PANSS. The PANSS positive symptom subscale is used to rate positive symptoms of schizophrenia across seven domains e.g. delusions, hallucinations, etc. The scores for each item are summed so that the minimum score is 7 (all symptoms are absent) and the maximum score is 49 (all symptoms are present and extreme). The PANSS negative symptom subscale is used to rate negative symptoms of schizophrenia across seven domains e.g. blunted affect, stereotyped thinking, etc. The scores for each item are summed so that the minimum score is 7 and the maximum score is 49. The PANSS general psychopathy symptoms subscale is used to rate symptoms that are not covered by the positive or negative subscales. This subscale covers 16 domains e.g. somatic concerns, anxiety, depression, lack of judgement and insight, etc. The scores for each item are summed so that the minimum score is 16 and the maximum score is 112.
